# Toward Co-productive Learning? The Exchange Network as Experimental Space

**DOI:** 10.3389/fsoc.2019.00036

**Published:** 2019-04-24

**Authors:** Rachel Matthews, Constantina (Stan) Papoulias

**Affiliations:** ^1^National Institute for Health Research (NIHR), Collaboration for Leadership and Applied Health Research and Care (CLAHRC) for Northwest London, Imperial College London, London, United Kingdom; ^2^Health Service and Population Research, King's College London, London, United Kingdom

**Keywords:** co-production, collaboration, action learning, reflexivity, patient and public involvement, quality improvement

## Abstract

Policy around patient and public involvement (PPI) in the production, design and delivery of health services, and research remains difficult to implement. Consequently, in the UK and elsewhere, recent years have seen a proliferation of toolkits, training, and guidelines for supporting good practice in PPI. However, such instruments rarely engage with the power asymmetries shaping the terrain of collaboration in research and healthcare provision. Toolkits and standards may tell us little about how different actors can be enabled to reflect on and negotiate such asymmetries, nor on how they may effectively challenge what count as legitimate forms of knowledge and expertise. To understand this, we need to turn our attention to the relational dynamic of collaboration itself. In this paper we present the development of the Exchange Network, an experimental learning space deliberately designed to foreground, and work on this relational dynamic in healthcare research and quality improvement. The Network brings together diverse actors (researchers, clinicians, patients, carers, and managers) for structured “events” which are not internal to particular research or improvement projects but subsist at a distance from these. Such events thus temporarily suspend the role allocation, structure, targets, and other pragmatic constraints of such projects. We discuss how Exchange Network participants make use of action learning techniques to reflect critically on such constraints; how they generate a “knowledge space” in which they can rehearse and test a capacity for dialogue: an encounter between potentially conflictual forms of knowledge. We suggest that Exchange Network events, by explicitly attending to the dynamics and tensions of collaboration, may enable participants to collectively challenge organizational norms and expectations and to seed capacities for learning, as well as generate new forms of mutuality and care.

## Introduction

This article aims to contribute to a literature that examines the complex, uneven, and often contradictory dynamics underpinning participatory approaches to health research and service development. The involvement of patients, carers, and the public in healthcare research and quality improvement is an established policy imperative in the UK and internationally, for example in Canada, Australia, and Scandinavia (Ministry of Health and Care Services, 1999; Boivin et al., 2010; Farmer et al., 2018). In the UK, the National institute for Health Research as well as other major health research bodies expect plans for such involvement (known as Patient and Public Involvement–PPI) to be detailed in all applications for research funding^1^. This imperative rests on a claim that when people are involved in the development of treatments and healthcare practices and in decisions around their provision, outcomes, and treatment relevance can be improved while care failures and research waste can be minimized (Carter et al., 2013; Chalmers et al., 2014). However, policy around PPI in the production, design, and delivery of health services remains difficult to implement, in part because the demand for involvement is underpinned by different and potentially conflicting rationales conceptualizing its nature, aims, and values (Knaapen and Lehoux, 2016). For example, the claim that patient involvement will increase the relevance of treatments and services points to both a potential democratization of health services in accordance with citizen needs and values, and a managerialist requirement for efficient services. The latter requirement can come into conflict with the former when it is employed to facilitate decommissioning in the guise of consumer choice (Beresford, 2003). It is also the case that a substantive incorporation of “lay” perspectives and insights into research and service redesign may necessitate too radical a transformation of what counts as legitimate forms of knowledge and expertise and may put into question the power relations which sustain such expertise (Rose, 2017). As a result, many initiatives are limited to an *ad hoc* or tokenistic level, where service users may, at best, have some input in refining minor aspects of the project (typically information sheets and promotion literature), but carry little weight in the overall shaping of healthcare interventions as well as in decisions about commissioning and healthcare provision more generally (Ocloo and Matthews, 2016; O'Shea et al., 2017). Furthermore, there is considerable evidence that those most affected by health inequalities (such as black and minority ethnic populations, older and younger people) remain “seldom heard” in PPI initiatives (Beresford, 2013; Dawson et al., 2018). In this context a growing body of literature seeks to improve practice by identifying barriers and facilitators of PPI; generating evidence of the impact of PPI in research and quality improvement and producing roadmaps for its successful implementation (The PiiAF Study Group, 2014; Staley, 2015; Stocks et al., 2015; Wilson et al., 2015; Staniszewska et al., 2017).

While the emergence and proliferation of this literature points to a desire to stabilize and clarify what might constitute “good PPI,” some see this proliferation as contributing to form of “busywork” which fails to challenge the power asymmetries through which broader socio-cultural inequalities enter and shape the terrain of collaboration in research and healthcare provision (Madden and Speed, 2017). Others have argued that clinicians' and managers expectation that patients' unique perspective can provide solutions to particular problems in research or healthcare provision, may perpetuate an extractive logic of passive patients as resources to be mined (Gilbert, 2018). Furthermore, while the emotional force of patient narratives is frequently noted, there is little evidence that such narratives alone can have a lasting transformative effect on clinicians' and researchers' practice (Adams et al., 2015). In this context, guidelines, toolkits, and standards, although helpful for the planning and budgeting of involvement activities, may tell us little about how different actors can be enabled to reflect on, negotiate and, where necessary, challenge such power asymmetries within a process of knowledge generation. A mechanistic focus on barriers and facilitators does not necessarily advance our understanding of the relational dynamic of collaboration in which different and potentially conflictual forms of knowledge are brought into play (Tritter and McCallum, 2006). To address this, a growing number of studies of PPI make use of ethnography and observation to explore how organizational cultures may constrain collaboration. For example the rigidity and episodic nature of steering group meetings as well as their formal apparatus (agendas, reports, and minutes) may disallow the development of trust and shared habits of work (Martin and Finn, 2011). Furthermore, existing disciplinary and institutional frameworks may work to constrain, neutralize, and appropriate patient voices (El Enany et al., 2013; Renedo et al., 2018).

Yet, while ethnographic methods have provided powerful analyses of what fails to happen in many PPI initiatives, there have been fewer attempts to analyse how alternative forms of collaboration between healthcare professionals and patients can adjust the relational choreography of PPI encounters and open up their transformative potential (Aveling and Jovchelovitch, 2014). Such attention would require a shift away both from listing barriers and facilitators, and from anatomizing ever more precisely all the reasons why PPI initiatives fail to work. Rather, it would demand, we argue, paying more attention to *finding ways to enact collaboration differently*—and to analyzing how such collaboration might work, unevenly and asymmetrically, in practice. In this paper, then, we turn our attention to the development of the Exchange Network, an innovative endeavor which seeks to foreground and reflect on the dynamics of collaboration themselves and explicitly utilize them as a site of learning.

## The Exchange Network: an Experimental Space

The Exchange Network (henceforth abbreviated as EXN) is an experimental space which brings together diverse actors (researchers, clinicians, patients, carers, and managers) with the aim of attending to and transforming the relational dynamics of collaboration. Crucially, it does so by providing a shared environment which does not arise within a research or improvement project or as a mechanism of organizational oversight but subsists independently and at a distance from these. This distance is both geographic (meetings occur outside institutional spaces) and conceptual: the EXN aims to temporarily suspend the role allocation, timeframe, organizational demands, and other pragmatic constraints of research and improvement projects. We argue that in so doing, the EXN functions as a “knowledge space” (Elliott and Williams, 2008; Gibson et al., 2012): a site which potentiates a dialogic encounter between participants by negotiating the power relations which underpin this encounter.

In this paper, we provide a schematic account of the form, setting, iterative emergence, and key features of the EXN as it is currently organized, to give due attention to how a focus on its choreography might open up some of the—often obscured—aspects that help us understand what is at stake in PPI initiatives. We then proceed to analyse this choreography—and consider how and to what extent the EXN can negotiate the conflicting rationales animating PPI work and interrogate the power relations that underpin it.

Our account of the EXN is not a presentation of research findings. We have not conducted formal research on the workings of the network: rather, this paper seeks to critically elaborate the position of intimacy from which we both write: one of us (RM) is a healthcare professional who has been involved in the co-design of the EXN from its inception and is currently the custodian of the space; the other (CP) is a service user academic who joined the EXN as a critical friend over a period of 2 years. We write from within the choreography rather than analyse it from the outside—and this undoubtedly allows us to describe certain features and dynamics of the EXN at the same time as it makes us less aware of others. This article is offered as a token of our collaboration and as an invitation to engage with the generative logic of the EXN.

## Setting

The EXN emerged during the tenure of the NIHR Collaboration for Leadership in Applied Health Research and Care (CLAHRC) NorthWestLondon. The CLAHRCs are infrastructure grants specific to English regions and awarded on 5-year cycles by the Department of Health and Social Care since 2009. Their purpose is to expedite improvements in the national health service (NHS) on a local level through translation of research evidence into clinical practice and through establishing sustainable partnerships across a number of stakeholders (universities, health service trusts, commissioners, charities). Since the CLAHRCs are mechanisms set up to improve clinical outcomes in a region, the funder places strong emphasis on demonstrable involvement by service users and/or carers in that region. There are currently 13 CLAHRCs covering most regions in England; with each one bringing a distinctive focus to this objective, both in terms of the specific health services they concentrate on and in terms of the kinds of partnership they favor. CLAHRC North West London focuses on developing emergent quality improvement and evidence translation models capable of acknowledging the complexity of healthcare systems. This means that such models work by eschewing solution based interventions, tolerating uncertainty, and engaging multiple actors in improvement efforts (Reed et al., 2014, 2018). In adopting these models, this CLAHRC could be said to foster a collectivist culture privileging the relational and contingent aspects of knowledge production and improvement (Renedo and Marston, 2015). This orientation provided a hospitable setting for experimentation with innovative forms of patient and carer involvement.

## Rationale and Phased Emergence

The EXN developed through a collaboration between service users and carers with PPI leads and managers working together through CLAHRC North West London funding. An initial team of 13 people came together through a series of externally facilitated meetings to explore possibilities for an alternative working practice. Participants had been involved in earlier CLAHRC improvement projects and “effective patient” training initiatives and shared a professed dissatisfaction with what they perceived as the tokenism and top-down dynamic of customary PPI practice. The group were encouraged to work in an experimental manner, embedding the ethos of CLAHRC North West London quality improvement models and resisting pressure to make assumptions about what shape such a space could take. Broadly, the EXN developed in three phases through exploratory, design and testing work (Table 1). Initial “exploratory” meetings between 2013 and 2014, tried out a plurality of organizing values for the future design of the network. Chief among these was an enduring orientation toward an ethos of co-production, as this was expressed in a set of principles developed by the National Endowment for Science Technology and the Arts (NESTA), a London based foundation which favors participatory approaches to public sector innovation. These principles, themselves an adaptation of the values laid out in Edgar Cahn's democratizing model of co-production, include a recognition of people's different assets; the development of reciprocal relationships between professional and lay members of a team; an emphasis on establishing networks; the development of activities to fit people's skills; an orientation toward personal development (Cahn, 2000; NESTA, 2012). The team recognized that adopting this model in its entirety might be beyond their capacity at present and settled instead on one of the principles: the development of reciprocal relationships in collaborative spaces. The proposed name of the emergent formation—initially “exchange group” later evolving to “exchange network”- signaled a similar attentiveness and, in particular, the ambition of the group to develop a certain practice of reciprocity: “exchange” pointed to a give-and-take which would define the nature of the workshops; “network” testified to the open, fluid and inclusive nature of the space.

**Table 1:** Evolution of the exchange network.

	**Explore phase**	**Design phase**	**Test phase**
Membership	*n* = 13	*n* = 13	*n* = 65
Meetings	*n* = 3	*n* = 3	*n* = 17
Meeting design	Chaired or facilitated	Facilitated	Co-facilitated
Structure	Agenda	Question based agenda	Semi-structured
Content	Imposed	Negotiated	Negotiated, co-designed
Sample content	Ground rules	Roles and responsibilities	Involvement as an aspect of mental health recovery
Influential features introduced following reflection	Third party facilitator	Using questions to frame discussions	Action Learning
			Dialogic process
			Explanatory materials
			Pre-attendance interviews
Integration with NIHR CLAHRC NWL programme	No	No	Yes, through collaborative learning events and improvement leader fellowship
Connections outside of NIHR CLAHRC NWL	No	Yes (new design group members)	Yes e.g., (NIHR CLAHRCs South London, North Thames, West Midlands, CCG representative, PhD students, NHS clinicians, community contacts)

In 2014, the initial team, having established potential organizing principles and aspirations for the group, proceeded to a second, “design” phase, in order to determine how to make the network “live.' This meant parsing out of the initial themes a coherent infrastructure and mechanisms capable of forming and sustaining the group (membership, learning methods, payment, evaluation strategy). In 2015, the actualization of the network began through a “testing” phase, which consisted in the delivery of regular planned events—quarterly meetings of 4–5 h in duration, iteratively adjusted through plan-do-study-act (PDSA) cycles (Taylor et al., 2013) (Table 2). During this phase, membership expanded from the initial 13 to 65 through engagement of broader audiences in various CLAHRC collaborative events and other networks. To date (February 2019) 17 such meetings have taken place.

**Table 2:** Test phase plan-do-study-act-cycles—testing form and function.

**No**	**Date**	**Title**	**Complete cycle?**
1	04/02/2015	Acceptability of membership and asset mapping forms	Yes
2	28/04/2015	Can an early planning conversation with Exchange Network Members generate new ideas for a research team?	Yes
3	06/05/2015	How do we extend the network?	No
4	17/09/2015	Welcoming new members and introducing group tasks	Yes
5	19/11/2015	Welcoming new members and introducing group tasks–repeat	No
6	17/03/2016	Welcoming new members and introducing group tasks–repeat	Yes
7	16/06/2016	Activating new members to contribute	Yes
8	01/09/2016	Activating new members to contribute–repeat	Yes
9	24/11/2016	Activating new members to contribute–repeat	Yes
10	15/06/2017	Introduce new co-facilitator	No
11	14/06/2018	Introduce new co-facilitator	Yes

In what follows we focus on particular aspects of EXN development in order to indicate how it attempts to re-orient involvement practice toward a horizon of co-production.

## Current Format and Essential Features

Currently EXN meetings follow a two-part format: each event brings together 15–20 people who gather in an accessible community venue with good transport links. The EXN has no lead as such: however the group is currently administered by one of the founding members employed through CLAHRC funds (RM) who acts as its custodian and moderator. Before each meeting, members are invited to contribute topics or bring particular problems to the room. Each workshop starts in a large group before breaking into smaller sets of four to five. In the morning sessions, the group responds to one or more topics presented by a new or existing member (these vary from troubleshooting a research proposal, offering learning points from a completed project, discussing potential methods for involving patients and the public in proposed collaborations—see Table 3). In the afternoon, members in the smaller facilitated groups take it in turns to work on a specific challenge or issue reported by one amongst their number. These can be research or quality improvement related problems, or equally, issues relating to the work environment itself or the organizational cultures experienced by participants (for example experiences of racism or discrimination). At the end of each session the whole group comes together once more to reflect on learning for the day. These reflections are then used to shape and improve on ensuing meetings.

**Table 3:** Examples of “air time” use in the Exchange Network.

• How to be more influential with individuals who hold different power to them for example in a meeting or in a relationship
• Seeking knowledge and feedback on past action in order to inform future action for example successfully recruiting community health champions and then struggling to evolve roles in a meaningful way
• How to obtain information and gain insight without making people defensive
• Sharing embryonic plans to capture patient experience in initiatives to improve care
• Reflecting on the challenges of being a carer for children with long term conditions
• Reflecting on the discrimination experienced as a carer from a BME background
• Practicing how to construct questions that enable people to work out “what matters to them” rather than just asking “what's the matter”?
• Seeking feedback on draft ideas to recruit lay advisors
• Sharing information about contacts and events
• Sharing relevant information and intelligence about local, regional, and international initiatives

EXN meetings make use of action learning sets to activate shared problem solving. Developed by Reg Revans between the 1940s and 1960s in the context of management education, action learning sets are a set of techniques which aim to enable members of an organization to address emergent complex problems by coming together to learn from each other (Revans, 1982). While there are different orientations in action learning, their shared characteristics include foregrounding the learner; privileging experiential learning over established “expertise” (in Revans' terms “insight” over “programme”); and making use of social interaction for the generation of such learning. Ultimately, the aim of action learning techniques is to be generative—while they have a pragmatic, problem solving focus, they mobilize action on problems through the sustained production of open questions, rather than the provision of answers (Pedler et al., 2005). Speakers present a problem as a starting point and discussion seeks to unpack its premises and, where possible, to challenge the speaker's assumptions about its intractability. In this way action learning proposes a move beyond tackling specific problems, to creating and seeding the capacity for “insight,” that is, the ability to “learn how to learn.”

These action learning techniques are selectively deployed in EXN meetings: groups are fluid and participants are not expected to turn up every time or to report on progress. Rather than focusing on problem solving, meetings focus on the relational space itself and situate action learning within a broader dialogic/reflexive envelope. This means that participants are not directed to specific actions or held to account for performance. Instead participants are encouraged to challenge their habitual ways of positing and engaging with problems while also focusing on the group dynamic in the room as itself a site of learning. They are encouraged to actively listen by observing the impact verbal and non-verbal communication has on them and the effect they have on others. In doing so participants begin to identify how defensive patterns might circulate across the group and how these patterns may themselves produce and perpetuate opposition and conflict. The use of co-facilitation in the workshops embeds this active listening as the facilitators support the group in reflecting on the dynamics of the encounter. Co-facilitation also supports critical reflection on the nature and impact of individual facilitator styles through the interaction between facilitators. The smaller afternoon groups more closely resemble typical action learning sets, with each participant taking it in turns to present an issue which the others will discuss, while resisting the impulse to give advice or propose a solution. Here different members may volunteer to facilitate, although this role is usually taken up by more regular and established members of the group or those who are more experienced in action learning techniques.

A feedback session which concludes each meeting ensures that the group assumes an active role in the ongoing iteration of a learning space by providing continuous evaluation: the feedback process, elicited through a further series of open questions, allows participants' observations and suggestions to adjust the workshop for consecutive meetings. Furthermore, “air time” issues are followed up where necessary with group emails and sharing of resources.

## The Exchange Network as a Space of Tension

The unique properties of the EXN reside in members' professed determination to move away from the structured approaches characterizing much of patient and public involvement (PPI) and engagement initiatives in both health research and health service delivery (for example, steering/advisory groups or committees, where “patient or carer reps” are included in professional teams) and the armature which sustains these (membership process, terms of reference, agendas, and minute taking). The process of moving away from these structured approaches to PPI is fraught however: in its successive stages, the evolution of the EXN testifies to an ongoing engagement and negotiation of the expectations associated with such structured approaches and with organizational expectations of “output” and a trying out of alternative solutions. Materials generated in the different phases of EXN work bear the traces of this negotiation—it is to these materials we now turn.

A tension between an emphasis on process vs. one on outputs became visible in the early phases of planning and is registered in the notes made during discussions in the exploratory workshops organized to guide the development of the group (Table 4). These notes suggest that the group shared an understanding of the importance of PPI in research and quality improvement, but what this shared understanding is, is never specified, except through an insistence that the group's perspective on PPI is different from and opposed to routine PPI practice and through a sense that their vision is embattled (“our vision is opposed”; “organizations…don't share our goals,” “[the group will be] a lifeboat for those who do get it”). Furthermore, the notes include several references to “value” and sketch out competing rationales for the purpose of patient and public involvement: value is perceived as economic benefit, as reduction of research waste, as influence on policy and health services commissioning, as patient activation, self- leadership and individual empowerment or as the “unfreezing” of individuals' assets. Here, distinct forms of social, economic, and personal benefit rub against each other and are held together without resolution, thus posing the question of how these differing and potentially contradictory aspirations can be reconciled.

**Table 4:** Exploratory phase.

**WHAT PROBLEM ARE WE TRYING TO SOLVE?**
• More of the “good” stuff'—rapid spread the message of engagement and exporting a culture that has developed through CLAHRC NWL
• Create a product that is useful to a range of people
• Create an impact on health and social care—e.g., commissioning
• Realize individual potential—unfreeze assets
• Realize economic benefits, create value, reduce waste—wide benefits
• Desire to influence what happens outside of CLAHRC (Health and Well-being Boards/local politicians/providers & CCG's/scope of influence
• Increase diversity—be able to sit with a range of views, create a spirit of inquiry. Ask more questions
**WHAT MAKES THE EXCHANGE GROUP DIFFERENT?**
• Relationships formed over an extended period
• Trust—N.B. types of trust
• Regular contact and events
• Spaces created—Fellowship, activation, equal partnerships, catalyst
• Time—allocated to work together, positive encouragement to think and reflect
• Diverse opinion and perspectives
• Relationships
• Authenticity
• Self-leadership
• Learning culture
• Community of practice—networking, connectivity
• WITH and BY, not TO, and FOR
**WHY ARE WE CREATING THE EXCHANGE GROUP?**
• To take risks
• A lifeboat for people who do get it
• Connection to a range of tools, support from others
• To provide nourishment and energy
• Networking
• Meet emotional and rational needs
• Compassion/courage/challenge
• Say what others want to be can't
**WHAT ARE THE RISKS?**
• Feels like too big a task—break it down into manageable goals
• Need to interact with organizations and individuals who don't share our aims/goals
• Our vision is opposed
• Regression to old culture

In the subsequent “design” phase the initial ambitions are narrowed down in an attempt to sketch out potential outputs for the group. Notes from this phase show that the question of measuring success in concrete terms comes to the fore (Table 5). Here, the initial assertion of a shared vision becomes specified as a demand for a membership strategy, “asset mapping” is proposed as a way of facilitating the “unfreezing of assets,” while considerations of reward and payment come to the fore and potential criteria for the measurement of success are suggested (for example, members propose delivering satisfaction surveys, tracking increase in membership, recording social media influence, developing training, or fundraising).

**Table 5:** Design phase.

**EMERGING QUESTIONS**
1. A membership strategy (who can join, how do they join, what support can be expected, and how do members leave?)
2. How do we identify and share individual skills, knowledge and experience (asset mapping)?
3. Where do we start with principles of recognition, reward, and payment?
4. How will the “exchange mechanism” work? What are the offers or the menu of opportunities?
5. How will we capture learning and share it with others?
6. How will we demonstrate and measure success?
**SUGGESTED SUCCESS MEASURES**
1. Increased number of members
2. Increased number of invitations to external workshops/meetings/seminars/groups
3. Increased funding
4. Increased outreach
5. Increased menu of opportunities
6. Internal and external collaboration between Exchange Network members
7. Completing tasks, goals and objectives—with accurate record-keeping
8. E-bulletins
9. Shared stories, tips, and tricks
10. Publications, articles, and features
11. Social Media Presence—trending hashtags
12. Impact (for individuals and sectors) measured by satisfaction survey and confidence and ability to voice opinions
13. Involvement with local government—Health and Well-being Boards
14. Becoming involved with CCGS and being “quality” control representatives
15. Certificates or other recognition
16. Training courses—co-training
17. Community and public celebration events

In the next, “testing” phase, as regular workshops start running, the structures, and measures drawn out in earlier phases are tried out, discussed and adjusted. For example, the membership forms and an associated “asset mapping” exercise proposed in early workshops are initiated, but participants eventually abandon them stating that it was not clear why that information needed to be collected and how it might be useful for the network. |In the absence of such documents and role specifications, alternative ways of explaining what the network is and how it operates are sought, so that new members could be attracted, and retained. While induction documents with information about the history and background to the EXN were initially drafted, these were then abandoned, as participants found that such information failed to reduce discomfort. Furthermore, it was felt that such documents were not useful in communicating the flavor and purpose of the meetings and the way in which each encounter foregrounded the quality of relational dynamics. In the place of induction documents, an informal one-on-one induction was introduced starting in 2016. This consists in brief pre and post-meeting conversations between new members and one of the co-facilitators. These conversations, also modeled on action learning set principles, do not focus on providing information on the meetings, but concentrate instead on asking prospective participants to speak of their expectations, anxieties and possible concerns about them. Additionally, in 2017, facilitators produced a brief document to serve as a more formalized induction to the network (see Supplementary File). This document affirms the orientation of the EXN around co-production, introduces the “operating principles” of dialogic learning and presents “the ladder of inference” a tool adapted from action science work on organizational change (Argyris, 1990). The ladder surfaces how tacit beliefs, assumptions and values may shape and determine action and invites the viewer to attend to and transform habits of selective listening. The presence of the ladder on the induction sheet thus visualizes the EXN purpose (a move from a defensive to an open practice of listening) as a simple, memorable image. The document, consisting of two sides of an A4 sheet, is currently distributed to all participants at the beginning of each meeting while facilitators reiterate the principles outlined there, so that all participants, whether regular, occasional or new, can start each workshop on the same footing.

While the EXN enacts parity and active listening in its principles, facilitation, and induction materials, it remains uncertain how far these can reach, or to what extent they can challenge institutional priorities and the logic of performance management in which these are embedded. Feedback comments collected during sessions, while necessarily brief, typically reiterate participants' appreciation of the reflexive approach and open-endedness of EXN workshops and of their difference from the culture of performance and scrutiny typical of the organizations they inhabit. However, participants may on occasion report some anxiety or discomfort with the absence of concrete measurable outputs or follow up action on the problems they may bring. Furthermore, this same open-endedness and fluidity of the meetings also means that on occasion clinicians and researchers may tap into the group in a tokenistic or extractive manner and see it as no different than an advisory or PPI group which is there to serve researchers' predetermined outcomes (for example by improving information sheets and consent procedures).

## Discussion

We have suggested that in proposing to concentrate on the relational dynamics of collaboration the EXN is poised between the undoing and reassertion of organizational values and priorities. In this discussion section, we further open out some of the key arenas through which such possibilities, asymmetries, and tensions take form.

### Techniques, Tacit Knowledges, and Rituals of Inclusion

Typically, PPI work in research and quality improvement is constituted through a series of regular patterned meetings set up as advisory or steering groups. These meetings, while exercising oversight for particular projects, also serve to socialize members to organizational cultures: they install and validate institutional codes, priorities, and appropriate behavior via the circulation of communicative objects (agendas, minutes, or reports) (Schwartzman, 1989). Such objects work to bind new actors into an organizational culture, its timelines, conventions, and expectations, thus enacting a professionalization of outsiders. In so doing, they arguably neutralize the potential of service user knowledges and perspectives to disrupt institutional habits (El Enany et al., 2013; Croft et al., 2016). By contrast, EXN workshops make use of a plurality of techniques borrowed from action learning and dialogic pedagogies in order to cleave apart such organizational habits and interrogate the tacit knowledges and values these embody. The decision to host the EXN in an accessible community venue embodies this intention to relax organizational expectations and allow for a proximity to community concerns. The adoption of an open-ended quality improvement model for EXN events ensures that such events are never decided in advance and brings to the fore their constitutive dynamics instead of rendering these invisible in favor of focusing to the “business-at-hand.” EXN communicative objects, such as the induction sheet, also work to bind new members to the culture of the group: the document's references to group principles, to dialogue and to the ladder of inference provide anchor points—what the original patient facilitator called a “structure and container”^2^—for the meetings. The circulation of the document, the act of reading aloud and re-asserting its principles at every meeting thus acquire a ritual function: in re-affirming the group purpose, they generate a sense of belonging and sustain relational inclusivity (Clarke et al., 2019). These acts explicitly reiterate this culture at each meeting as the ongoing work of suspending habit and set it up as *something contingent, that is, as what is produced anew in the present of each encounter* and is therefore dependent on new members' decision to act in a particular way (to produce open questions, to challenge assumptions and so on).

### Action Learning: Compliance and Transformation

The use of action learning sets in the EXN is far from unique in the context of healthcare organizations. Indeed, there is currently a global proliferation of action learning workshops for healthcare professionals and teams (Chivers, 2005; Attwood, 2007; Mathews et al., 2017) and it can be argued that, since the initial development of action learning sets took place in English state hospitals during the 1960s, Revans' work emerged in response to the specific institutional dynamic of the NHS and as a way of harnessing knowledge and resources from staff in order to improve their ability to function within its organizational complexity. Revans and his successors have written extensively about the ability of action learning techniques to increase efficiency by managing and regulating the particular kinds of risk and anxiety experienced by the NHS workforce (Revans, 1982). In this context some have argued that the increased investment in action learning workshops by healthcare organizations, testifies to their *effectiveness as a technology of compliance* which aims to ensure that staff at all levels willingly internalize and embed organizational priorities. This internalization means that staff may come to see the implementation of policy imperatives as a marker of personal empowerment and efficacy (Brook, 2010). However, more recently developed critical action learning approaches have broadened their reach from working with the experiential knowledge of participants to reflecting on and calling into question the assumptions and values which underpin such knowledge (O'Neil and Marsick, 1994). Unlike Revans' original model, critical action learning does not primarily focus on problem solving and on actions emerging as a consequence of group meetings. Rather, it concentrates on the meeting itself, and the affective and communicative dynamics through which a group is constituted. Here, the role of the facilitator or coach is central in ensuring that such dynamics are surfaced as an object of learning. The ethos of these reflexive models resonates in the interactions characterizing EXN meetings. EXN workshops draw attention to facilitation, engagement with the relational climate in the room and the quality of interactions among participants. In so doing they focus on the power dynamics and tensions which constitute the process of collaboration. Since EXN meetings do not operate as conventional action learning sets, there is no expectation that participants will produce an action plan and report on progress in subsequent meetings (although any such report is welcomed). Instead, meetings make use of Argyris' ladder of inference and other tools to identify how established institutional habits may define participants' responses to each other and to loosen the grip of such habits so that participants may develop a capacity for open listening. In some forms of critical action learning, an interrogation of institutional habits can extend to an interrogation of organizational priorities and an understanding of how these may be shaped by and contribute to the perpetuation of wider socio-political inequalities (Marsick and O'Neil, 1999; Rigg and Trehan, 2004; Traeger, 2017). This level of criticality is not explicitly spelled out during EXN meetings, yet the insistence on forging relational literacy through an interrogation of institutional habits also entails an interrogation of how healthcare professional habits may marginalize lay or service user knowledge. In their cultivation of relational literacy and open listening, EXN meetings thus orient action learning principles toward an ethos of co-production, understood here as a critical focus on group dynamics and as building capacity for working with others.

### Patient Leadership as a Complex Form

A discussion of the EXN cannot sidestep the foundational role of patient leadership initiatives to its origins, development and facilitation style. Patient leadership work rests on the argument that the “lived experience” of patients can be repurposed to enact a form of leadership from below, and become a key resource for service improvement, in so far as it works in partnership rather than opposition to healthcare professionals. Its proponents privilege dialogic interaction and develop techniques for building leadership skills with patients while shunning the practices of what they call “the engagement industry” (which typically relies on consultation, patient feedback mechanisms and the installation of patient representatives in various steering groups). These practices, they argue, far from empowering patients, function to perpetuate institutional privilege, knowledges, and priorities, by delegitimizing patient voices (Gilbert and Doughty, 2012a,b; Gilbert, 2015). Patient leadership initiatives start by resituating the figure of the patient as an agent of organizational change rather than “a problem to be solved” and make use of experiences of distress as springboards for such change. In so doing, these initiatives can be said to counter organizational orthodoxies concerning the production of leadership qualities and to enable a paradigm shift toward a more “participatory medicine” (deBronkart, 2018). At the same time however, patient leadership initiatives explicitly borrow from self-leadership discourse and the conceptualizations of self-management that underpin it (Neck and Houghton, 2006). They thus rely on an underlying conceptualization of “the patient” as a resilient, self-regulating, self-efficacious agent of change. This conceptualization has been critiqued for its positioning of resilience as an individual psychological resource and minimizing the role of structural factors, such as social and economic inequalities, in conditioning people's sense of well-being and ill health and their sense of worth and agency (Miller and Rose, 2008; Friedli, 2012). In this reading, the figure of the patient leader becomes a social entrepreneur, whose sense of empowerment is atomized and therefore detached from a broader demand for organizational or social transformation (Miraftab, 2004; Carr, 2018). The discursive complexity of patient leadership initiatives raises the question of how far the dialogic ethos of the EXN may be undercut by participants' reliance on such models of atomized empowerment and of an autonomous, self-regulating subject,—models which may constrain or even run counter to the principles of interdependence, and collaborative exchange upon which these initiatives are built (Renedo and Marston, 2015).

### From Space of Tension to Experimental Space

While some of the founding principles and action learning techniques of the EXN may testify to a proximity to institutional and managerial rationalities and to atomized models of self-leadership as self-management, the experimental aspects of the workshop events through which the network is enacted may also be poised to unsettle these. By attending to EXN workshops as experimental spaces we borrow from Fitzgerald and Callard's work on such spaces as locations of a certain unanticipated productivity (Fitzgerald and Callard, 2015). The authors' work on what they call “experimental entanglements” stems from their autoethnographic exploration of the dynamics of collaboration in their own interdisciplinary work across the social sciences and the neurosciences. In carrying out this work they resist the temptation of denouncing collaboration as the subjugation of different actors by powerful institutions and their priorities. At the same time they refuse to imagine that reciprocity and mutuality between unequally placed “partners” is possible (Callard and Fitzgerald, 2015). Instead they invite us to acknowledge that all collaborative endeavors are shaped by power asymmetries and institutional priorities which may be intractable. Yet they also suggest that such asymmetries do not completely determine the outcomes of collaborative work. They thus propose that we think of collaborative spaces as experimental spaces, by which they mean encounters which can give rise to something new (connections, networks, findings) which is irreducible to their constituent parts and to their intended outcomes. An experiment is a generative space: it may arise within controlled conditions, but its outcomes cannot be anticipated or contained by these conditions. Attending to the logic of the experiment means considering how the workshop-events through which the EXN is actualized *cannot be decided in advance* or controlled through their institutional constraints. It means acknowledging that the EXN workshops are not about creating a level playing field but a “generative space” in which patients, clinicians, commissioners managers, and carers may invent new relational possibilities (Filipe et al., 2017). The emergent qualities of such a space may broaden our expectations of the forms that citizen participation in healthcare development can take.

## Concluding Remarks

We have suggested that by focusing on the relational aspects of collaboration and on the way in which knowledge comes to be legitimized in such collaboration, the EXN can generate a learning space which holds a promise of transformative encounters between patients, carers, researchers, and healthcare professionals. However, in so doing, it is also poised between the undoing and the reinforcement of institutional scripts, and its potential to work as a transformational encounter is undecided. Because of this we have suggested that the logic of the experiment might provide a more promising approach to an analysis of the EXN, one which calls attention to the unpredictability of the encounters it puts into play, without losing sight of the unequal distribution of power in which such encounters are inevitably mired.

Furthermore, while EXN workshops rehearse collaborative practice, it is unclear how such practice might ripple outside the bounds of the workshops themselves and what effects such rehearsals might have on practice more generally. If the EXN is less about problem solving than it is about building capacity for an ethical and reflexive collaborative practice, where might an evaluation of this practice begin? How might we begin to track the extent to which experimental spaces such as the EXN generate new relations of care and whether they are able to attenuate institutional dynamics of exclusion? And how could such evaluation enable us to bring to visibility the kinds of emotional and relational labor required to initiate and sustain such a collaborative space?

Providing answers is beyond the scope of the present article as it is indeed beyond the scope of the experiment itself. We hope, instead, that the experiment that is the Exchange Network can continue to invite us to pose new questions.

## Author Contributions

All authors listed have made a substantial, direct and intellectual contribution to the work, and approved it for publication.

### Conflict of Interest Statement

RM is funded via the CLAHRC North West London, an award from the National Institute of Health Research UK which also provides the funding for the Exchange Network, the series of collaborative workshops described in the article. RM is also the co-facilitator and curator of the workshops. The remaining author declares that the research was conducted in the absence of any commercial or financial relationships that could be construed as a potential conflict of interest.

## Footnotes

^1^In this paper we are broadly following a distinction between involvement (patients actively involved in design and decision making in clinical research and quality improvement), and participation (patients taking part in research as participants). This distinction is laid out by INVOLVE, the UK national advisory group on public involvement in research (see https://www.invo.org.uk/find-out-more/what-is-public-involvement-in-research-2/).

^2^The phrase “structure and container” as well as the selective appropriation of action science techniques relate to the work of patient consultant Mark Doughty who developed these techniques in his earlier collaboration with David Gilbert, at the Centre for Patient Leadership. David Gilbert is currently the first patient director in the British NHS at Sussex Musculoskeletal Partnership (Central), while Mark Doughty is senior consultant in the Leardership and Organizational Development Team at the King's Fund, a think tank working on the health system in England.
