# Supplementary material for: Toward Co-productive Learning? The Exchange Network as Experimental Space
Source: Front Sociol. 2019 Apr 24;4:36. doi: 10.3389/fsoc.2019.00036 (PMC8022628; doi:10.3389/fsoc.2019.00036)

NHS
National Institute for
Health Research

Collaboration for Leadership in Applied Health Research and Care
Northwest London

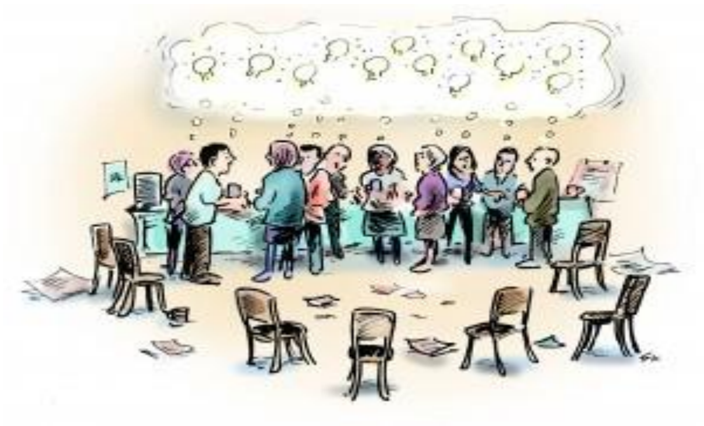

Welcome

What is the Exchange Network?

- A network of people from different backgrounds with a shared interest in improving healthcare, involvement, learning and leading together
- A facilitated space that acknowledges and seeks to equalise power differentials between patients, carers, service users, researchers and healthcare professionals in order to learn collaboratively
- A personal development opportunity

How did it develop?

The Exchange Network emerged from the experience of working with patients, carers and service users in the NIHR CLAHRC NWL programme from 2009 - 2013 who told us that they preferred a more collaborative approach that recognised and valued their individual skills and experiences. The network was developed **with** patients, carers and service users. It is underpinned by the principles of co-production in particular reciprocity and the desire to blur boundaries.

Purpose

The overall purpose is to promote high value, productive conversations and relationships that lead to new insights and ideas that network members can act on and take with them to apply in other situations.

What will it achieve?

We hope that members leave each meeting with something new. This may be a new contact, idea or reflection which they can use to progress areas they are interested in or activities they are responsible for.

Centre For *Patient Leadership*

The National Institute for Health Research Collaboration for Leadership in Applied Health Research and Care (NIHR CLAHRC) Northwest London is a partnership between Chelsea and Westminster Hospital NHS Foundation Trust and Imperial College London

Operating principles and practices for Exchange Network meetings

Participants are helped to clearly delineate between conversations based on the mind-set that gives rise to ***debate, discussion and dialogue***.

The focus of the Exchange Network is on **dialogue**. It is grounded in a mind-set that:

- Suspends judgement, comes with an open mind
- Deeply listens – to understand and find meaning and agreement
- Is focused on inquiring based on a spirit of discovery and genuine interest
- Uses powerful questions
- Is collaborative – working together towards common understanding
- Explores others and one's own assumptions, beliefs, attitudes for re-evaluation
- Acknowledge that others' thinking can improve one's own
- Is strength based and solution focused
- Is focused on discovering new options

To support the development of dialogue participants use a tool called ***The Ladder of Inference*** (Chris Argyris)***.*** This describes the thinking process we go through (often unconsciously) to get from a fact to an action or decision. Using the ladder supports participants in:

- Becoming more aware of your own thinking and reasoning (reflection)
- Making your thinking and reasoning more visible to others (advocacy)
- Inquiring into others' thinking and reasoning (inquiry)

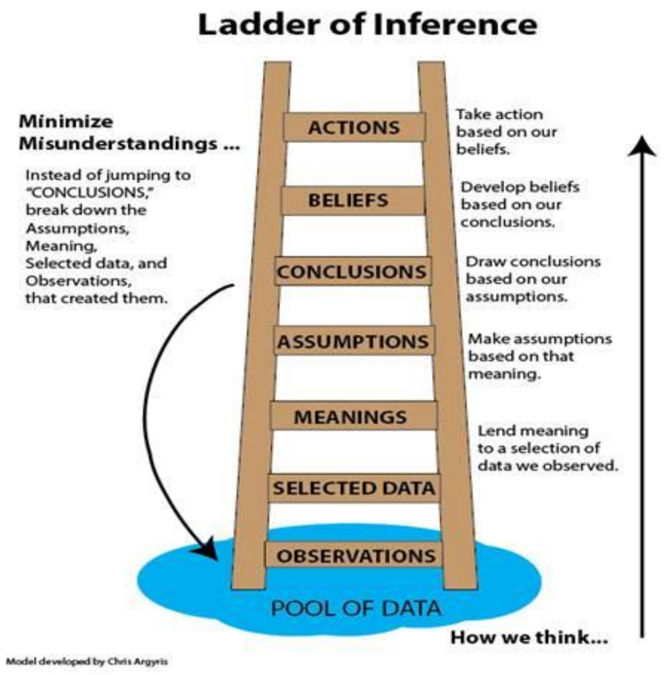

Supplement: Supplementary file 1 [file Data_Sheet_1.PDF]
